# How demoralization is related to trait resilience factors: a network analysis in a representative sample of the general population

**DOI:** 10.1186/s12888-025-07487-8

**Published:** 2025-10-13

**Authors:** Markus Ramm, Kathrin Schnabel, Johanna Jedamzik, Lara Jürgens, Jelena Gerke, Miriam Rassenhofer, Elmar Brähler, Gereon Heuft, Rupert Conrad

**Affiliations:** 1 Department of Psychosomatic Medicine and Psychotherapy, University Hospital Münster, Albert-Schweitzer-Campus 1, Münster, D-48149 Germany; 2 Department of Child and Adolescent Psychiatry/Psychotherapy, Center for Child Protection in Medicine in Baden-Württemberg, University Hospital Ulm, Ulm, Germany; 3 Department of Psychosomatic Medicine and Psychotherapy, University Medical Center of the Johannes Gutenberg University Mainz, Mainz, Germany; 4 Department of Medical Psychology and Medical Sociology, University of Leipzig Medical Center, Leipzig, Germany

**Keywords:** Control, Demoralization, General population, Network analysis, Resilience, Self-efficacy

## Abstract

**Background:**

Demoralization refers to a mental state of poor coping characterized by a loss of purpose and meaning, feelings of hopelessness and worthlessness, and suicidal ideation. The revised demoralization scale (DS-II) is among the most frequently used self-report measures. Recently, the psychometric properties and normative values of the DS-II-Ms (Münster version of the DS-II) were published, alongside a validation study linking it to depression and anxiety in the general population. This study investigates the relationship between DS-II-Ms scores and resilience, as well as well-validated trait resilience factors, specifically locus of control and general self-efficacy, using a network psychometrics approach.

**Methods:**

DS-II-Ms, Patient Health Questionnaire 2 (PHQ-2), Generalized Anxiety Disorder Scale 2 (GAD-2), Internal-External Locus of Control Short Scale-4 (IE4), General Self-Efficacy Short Scale-3 (GSE-3) and Brief Resilience Scale (BRS) were applied to a representative sample (*N* = 2401) of the German general population. A gaussian graphical model was estimated using a non-regularized algorithm to depict the unique connections between the measures.

**Results:**

DS-II-Ms was moderately associated with lower internal (and higher external) locus of control while being conditionally independent from BRS and GSE-3. Conversely, depression symptoms lack of interest/low mood were connected to resilience and general self-efficacy but conditionally independent from locus of control.

**Conclusions:**

Although the cross-sectional study design limits directional interpretation, our findings indicate that trait resilience measures have unique associations with demoralization and depression/anxiety symptoms, supporting the discriminant validity of the demoralization construct. Depressed and demoralized individuals might benefit from different therapeutical approaches, targeting specific resilience factors.

**Supplementary Information:**

The online version contains supplementary material available at 10.1186/s12888-025-07487-8.

## Background

The concept of demoralization was first introduced in 1961 by Frank who argued that individuals seeking psychotherapeutic support are often driven by a helpless inability to cope, and this mental state reflected the main therapeutic target across psychotherapeutic techniques [1]. In the context of medically ill patients, demoralization was defined as a mental state of poor coping, feelings of hopelessness and helplessness, and a loss of meaning and purpose in life [2]. It has been most frequently observed in patients with cancer and those in palliative care [3, 4], but in recent years, research has increasingly investigated demoralization across several physical and mental disorders [5]. This suggests that demoralization, viewed as a dysfunctional way of coping, may constitute a transdiagnostic concept [6]. A recent systematic review reported an overall prevalence of 24–36% in oncology and non-oncology, including those with mental health disorders [5]. Beyond its role in clinical populations, global stressors such as pandemic, can evoke feelings of help- or hopelessness in the general population [7]. Previous research has suggested that symptoms of demoralization during COVID-19 may be linked to suicide risk [8].

There is an ongoing debate in the literature about the distinction between demoralization and related constructs such as depression. In clinical populations, significant comorbidity with depression has been noted, with highly demoralized patients often also experiencing depression [9–11]. However, despite their clinical overlap, recent studies have demonstrated that certain features of depression (e.g. anhedonia, somatic and cognitive symptoms) are distinct from demoralization symptoms in both clinical [12, 13] and general populations [14]. Moreover, one of the most significant adverse outcomes related to demoralization is suicidality [15].

Acknowledging the crucial role of demoralization for public mental health, it becomes essential to improve our understanding of how individuals can be protected from developing demoralization, or, in other words, how they can become “resilient against demoralization”. In clinical populations, evidence regarding psychological factors that could be protective against demoralization is limited. Previous cross-sectional studies have revealed negative correlations between demoralization and post-traumatic growth, as well as sensemaking and benefit finding [16], resilience in tremor patients [17], resilience in prostate cancer patients [18] as well as self-efficacy and perceived control in cancer patients [19, 20]. Nevertheless, although our understanding of resilience’s role in mediating between predisposing factors and depression is continuously increasing [21–23], the precise pathways through which resilience and related resilience factors are associated with demoralization remain unclear.

According to the transactional resilience framework proposed by Kumpfer, when predicting resilience, multiple interacting resilience constructs including both factors and processes need to be considered [24]. When a resilient individual is challenged by a relevant stressor, a resilience process is initiated, during which internal resilience factors interact with environmental factors, fostering active coping and finally enabling the individual to bounce back with resilient reintegration [25]. An important self-characteristic of resilient individuals is an *internal* locus of control (in contrast to an *external* locus of control). According to Rotter’s locus of control theory, these individuals believe that events are under their control, leading to a hopeful and optimistic attitude in dealing with a stressor [26]. This sense of hopefulness and optimism contrasts Seligman’s concept of learned helplessness. Learned helplessness describes a mental state individuals may develop after they were repeatedly exposed to uncontrollable stressors, leading to the belief that one is powerless in changing an adverse situation [27]. Subjects who interpret the cause for not being able to control negative events as stable, global and internal are most likely to develop an expectation of generalized, chronic future helplessness accompanied by a lower self-esteem [28]. Helplessness represents the key symptom of demoralization while also being a risk factor for a decrease in motivation, passivity and finally depression. Furthermore, even if an individual believes that adverse situations are generally controllable, according to Bandura’s social cognitive theory, the expectations about one’s competencies in dealing with a particular situation (general self-efficacy; GSE) influence whether motivated coping is initiated and resilient reintegration is achieved [29]. This is in line with numerous studies that have associated self-efficacy with mental health problems [23, 30, 31].

In the present study, we aimed to comprehensively explore the specific interrelations between demoralization, depression and anxiety as well as resilience and resilience factors (including GSE and locus of control) to provide further support for the validity of the demoralization construct. For this purpose, network psychometric approaches are particularly suitable, as they allow to model complex (and unique) associations between psychological variables [32, 33]. Unlike traditional approaches that use latent variable analyses to identify shared underlying factors, network models aim to estimate a sparse network of conditional relations, thus identifying meaningful connections (“edges”) among manifest variables (“nodes”) [34].

As outlined by de Figueiredo, in patients with depression, the source of distress is internal and anhedonia as well as a lack of motivation are present, whereas moderately demoralized individuals give an external attribution to their distress and primarily experience a high level of subjective incompetence in coping while pleasure and motivation are preserved [35]. Thus, we hypothesized that both demoralization and depression (i.e. loss of interest/depressive mood) are conditionally dependent due to sharing a sense of helplessness, while they also have unique connections with resilience and resilience factors GSE and locus of control. We expected that, as depression may reflect the result of a repeated negative outcome of the complex resilience process, loss of interest/depressive mood is more closely related to resilience outcome itself while being conditionally independent from resilience factors such as GSE and locus of control. Moreover, as an internal state of generalized chronic helplessness, reflecting a low level of perceived control over internal and external stressors, may serve as a common factor among demoralization and internal resilience factors, we assumed that demoralization has unique connections with trait resilience factors, i.e. demoralization being conditionally dependent from locus of control and/or GSE, while it is thought to be conditionally less dependent (i.e. significantly lower edge weight) from resilience.

## Methods

### Participants

The data were collected between March and May 2022 by a demographic consulting company (USUMA, Berlin, Germany) as part of a comprehensive household survey in Germany. A detailed description of the data collection process has been provided previously [36]. In summary, Germany was divided into 258 distinct areas to ensure a representative sample reflecting the country's regional demographics. A multistage random sampling method was utilized to select 6192 households (of which 6188 were valid), creating a sample that is representative of the general population with respect to age and gender. The survey (for an English version see Supplement) was conducted through face-to-face interviews. Among the participants aged 16 and older, 2522 individuals (41.2%) agreed to participate. The final analysis excluded those under 18 years of age and participants with incomplete responses, resulting in a final sample size of *N* = 2401 participants.

### Measures

The DS-II-Ms is a German translation [36] of the original DS-II [37, 38] representing a self-administered assessment of demoralization consisting of 16 items, evaluated on a three-point rating scale (0 = never; 1 = sometimes; 2 = often). Items evaluate the frequency of experiencing distress following an inability to cope with stressors (e.g. “I feel trapped by what is happening to me”, “I do not cope well with life”) and a loss of meaning and purpose (e.g. “My life seems to be pointless”, “I feel hopeless”). Sum scores range between zero and 32. The DS-II-Ms demonstrated excellent internal consistency (Cronbach’s α =.94) within our study sample. In terms of dimensionality, previous confirmatory factor analyses suggested a one-factor model in the general population [36].

The Patient Health Questionnaire 4 (PHQ-4) is a short screening tool for assessing depression and anxiety symptoms [39]. The scale consists of two items of PHQ-9 [40], corresponding to the two key diagnostic criteria (“little interest or pleasure in doing things”, ”feeling down, depressed, or hopeless”; PHQ-2) of major depressive disorder as outlined in the Diagnostic and Statistical Manual of Mental Disorders, Fifth Edition (DSM-V). In addition, the scale incorporates two items from the GAD-7 [41] to screen for generalized anxiety disorder (GAD), focusing on “Feeling nervous, anxious or on the edge” and “Not being able to stop or control worrying” (GAD-2). Frequencies of the symptoms are rated on a 4-point rating scale ranging from 0 ("not at all") to 3 ("nearly every day") based on the individual’s experiences over the past two weeks. The sum scales of PHQ-2 and GAD-2 range from zero to six. Factor analysis confirmed the two-factor structure [42]. The German PHQ-4 (Cronbach’s α =.88) and its subscales PHQ-2 (α =.83) and GAD-2 (α =.79) revealed acceptable to good internal consistency within our study sample.

The Brief Resilience Scale (BRS) consists of six items (e.g. “I tend to bounce back quickly after hard times”, “I tend to take a long time to get over set-backs in my life”) rated on a 5-point Likert scale (from 1 = “strongly disagree” to 5 = “strongly agree”) [43, 44]. Higher mean scores on the BRS indicate a greater belief of an individual to be able to bounce back or recover from stress. Notably, this definition of resilience is distinct from other resilience concepts that e.g. target the resilience process. Cronbach’s alpha coefficient (α = 0.87) showed good reliability within our study sample. Previously, a method-factor model had revealed a superior model fit compared to the one-factor model [45].

The four-item Internal-External Locus of Control Short Scale**-**4 (IE-4) is a ultra-short self-report scale that was developed to measure both aspects of the personality trait locus of control according to Rotter [26] with two items each, i.e. internal (“e.g. If I work hard, I will succeed”) and external (“Fate often gets in the way of my plans”) locus of control [46]. Items are rated on a 5-point rating scale (from 1 = “does not apply at all” to 5 = “applies completely”). Mean scores for each subscale range from one to five. Confirmatory factor analyses showed a correlated two-factorial structure. Within our study sample, the IE-4 showed acceptable to good internal consistency for internal control (IE4-IC; α = 0.83) and external control (IE4-EC; α = 0.79).

The three-item General Self-Efficacy Short Scale-3 (GSE-3), corresponding to the German “Allgemeine Selbstwirksamkeit Kurzskala (ASKU; [47]), represents an economical measure of the personality trait “self-efficacy”. Self-efficacy refers to people’s beliefs in their own competence to solve a problem. Items (e.g. “I am able to solve most problems on my own”) are rated on a 5-point rating scale (from 1 = “does not apply at all” to 5 = “applies completely”). Mean scores of the GSE-3 range from one to five. Confirmatory factor analyses strongly supported a unidimensional factor structure [47, 48]. The GSE-3 exhibited excellent internal consistency within our study sample (α = 0.90).

### Statistical analysis

Primary analyses comprised the examination of convergent and divergent validity of the DS-II-Ms using bivariate correlations as well as network models.

Analyses were carried out using IBM SPSS® Statistics Software (version 28.0, IBM, Armonk, NY) and R version 4.3.1 [49]. For all analyses, critical *p* was set to 0.05.

A Gaussian Graphical Model (GGM) network was estimated to model the interrelations among DS-II-Ms, PHQ-2, GAD-2, BRS, IE4-IC, IE4-EC and GSE-3. Although previous factor-analysis showed a two-factorial model is superior to a one-factorial model [45], for reasons of interpretability the BRS is here included as a unidimensional measure.

Estimation of the GGM network based on the non-regularized *ggmModSelect* algorithm implemented in the bootnet R package (version 1.6; [33]). In case of large data sets in which the number of observations greatly exceeds the number of dimensions, non-regularized algorithms have shown to be superior to regularized algorithms (e.g. Graphical Least Absolute Shrinkage and Selection Operator with EBIC model selection; EBICglasso; [50]) in terms of sensitivity (i.e. high rate of identifying true network edges) and specificity (i.e. low false inclusion rate of edges; [51, 52]). Based on a correlation matrix computed by “cor_auto” function of qgraph R package, the *ggmModSelect* algorithm first generates a set of network structures by varying the LASSO tuning parameter, which are then re-estimated using non-penalized maximum likelihood (i.e. setting the LASSO tuning parameter to zero). Non-regularized estimates are used to optimize the EBIC. The *ggmModSelect* algorithm optimizes EBIC by stepwise adding and removing edges.

The resulting network presents the associations (“edges”) between two measures (“nodes”) as conditional effects (“edge weights”), relating to the direct effect between two nodes after controlling for the influence of all other nodes in the network. The thickness of the edge shows the strength, and the color indicates the direction (purple = positive; red = negative) of this association.

Node strength (i.e. the absolute sum of weights of all connections between one node and all others) as the most common and reliable centrality index is reported.

Bootstrapped difference tests based on 95% confidence intervals (CI) intervals using bootnet R package (version 1.6; [33]) were performed to examine differences in edge weights and node strengths.

Stability of node strength and edge weights were examined using a case-dropping bootstrap procedure with 10.000 samples. Correlation stability (CS) coefficients indicate the percentage of the sample that can be dropped to maintain, with a 95% CI, correlation values of r ≥ 0.7 between the sample’s networks estimates and the bootstrapped sample’s estimates. This index should be above 0.25, preferrable above 0.5 [33].

## Results

The research sample comprised 50.1% males, 49.7% females, and 0.2% non-binary. The mean age was M = 49.78 years (SD = 17.25), with a range from 18 to 96 years. Mean values and standard deviations of the included measures, as well as manifest bivariate correlations, are shown in Table 1. Overall, correlation coefficients ranged between “moderate” and “strong”. DS-II-Ms showed strong bivariate correlations with PHQ-2 and GAD-2, whereas BRS, GSE-3 and IE-4 moderately correlated with DS-II-Ms, which were comparable to the correlations observed for PHQ-2.

**Table 1:** Mean and standard deviations as well as Pearson’s correlation coefficients for included measures

	**M**	**SD**	**DS-II-Ms**	**PHQ2**	**GAD2**	**BRS**	**IE4-IC**	**IE4-EC**	**GSE-3**
DS-II-Ms	3.76	5.57	1	.70**	.67**	-.49**	-.48**	.40**	-.45**
PHQ-2	0.77	1.18	.70**	1	.77**	-.49**	-.45**	.40**	-.45**
GAD-2	0.65	1.10	.67**	.77**	1	-.46**	-.41**	.38**	-.40**
BRS	3.55	0.81	-.49**	-.49**	-.46**	1	.55**	-.47**	.67**
IE4-IC	4.13	0.77	-.48**	-.45**	-.41**	.55**	1	-.40**	.69**
IE4-EC	2.34	0.88	.40**	.40**	.38**	-.47**	-.40**	1	-.37**
GSE-3	3.97	0.77	-.45**	-.45**	-.40**	.67**	.69**	-.37**	1

*M* Mean, *SD* Standard deviation

**Highly significant (p < 0.001)

### The role of demoralization within a psychopathology and resilience network

**Fig. 1:**
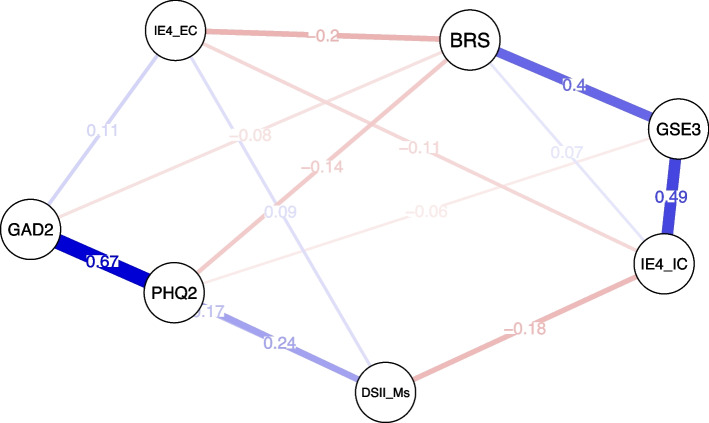
Partial correlation network showing the association between Demoralization Scale-II (DS-II-Ms), Brief Resilience Scale (BRS), General Self-Efficacy Short Scale-3 (GSE-3), Internal-External Locus of Control Short Scale (IE4-IC, IE4-EC), Patient Health Questionnaire 2 (PHQ-2) and Generalized Anxiety Disorder Scale 2 (GAD-2). Positive associations are displayed in purple, negative associations are red

**Fig. 2:**
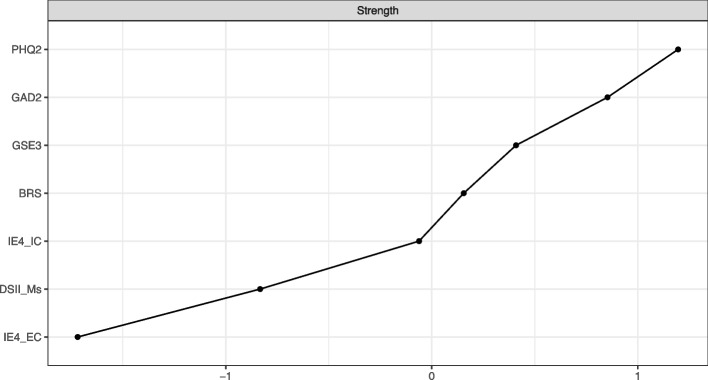
Z-standardized node strengths of Patient Health Questionnaire 2 (PHQ-2), Generalized Anxiety Disorder Scale 2 (GAD-2), General Self-Efficacy Short Scale-3 (GSE-3), Brief Resilience Scale (BRS), Internal-External Locus of Control Scale (IE4-IC, IE4-EC) and Demoralization Scale II (DS-II-Ms), ordered by strength. Node strength represents the absolute sum of a nodes’ edge weights

**Fig. 3:**
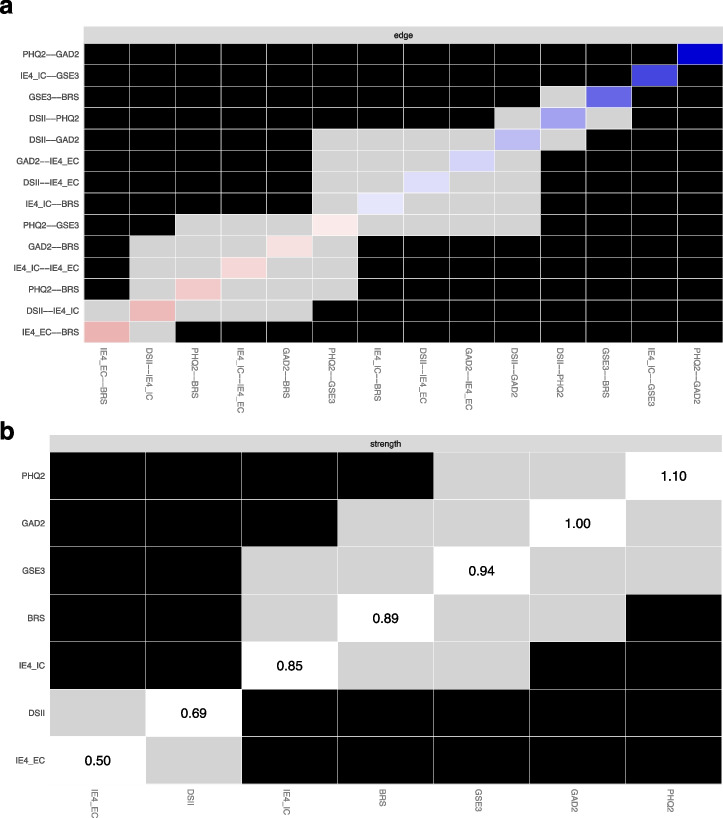
Bootstrapped difference tests (with α = 0.05) between non-zero edge weights (**a**) and node strengths (**b**) in the estimated network. Gray boxes indicate that two edge weights do not significantly differ while black boxes reflect a significant difference. Colored boxes in the edge weight plot represent the color of the edges in the network structure plot. White boxes indicate the unstandardized node strength

The network was made up of 7 nodes and network density was high, i.e. 14 non-zero edges out of 21 possible connections, representing 66.67% of all possible connections (Fig. 1). Figure 2 presents the centrality parameter “strength”, showing that PHQ-2, followed by GAD-2, were the most influential nodes (i.e. highest sum of absolute edge weights) in the network, whereas IE4-EC, followed by DS-II-Ms, had the lowest impact. As outlined by Fig. 3b, among resilience measures, GSE-3 (95% CI 0.37–0.62), BRS (95% CI 0.21–0.47) and IE4-IC (95% CI 0.17–0.53) exhibited significantly greater connection density (i.e. higher impact on the network) compared to IE4 external control (Figs. 2, 3). Most importantly, the node strength of DS-II-Ms was significantly lower than those of all other measures except IE4-EC.

As shown in Fig. 1, the strongest connections in the network were observed between GAD-2 and PHQ-2, GSE-3 and IE4-IC, as well as BRS and GSE-3. In contrast, DS-II-Ms was significantly less strongly connected to PHQ-2 (95% CI 0.29–0.51) and GAD-2 (95% CI 0.41–0.69) compared to the association between PHQ-2 and GAD-2. Similarly, edge weights DS-II-Ms – IE4 were significantly lower than edge weights of the resilience connections BRS – GSE-3 and GSE-3 – IE4-IC (Fig. 3a).

In sum, the results are in line with theoretical assumptions that the strongest associations are among trait measures of resilience (BRS) and resilience factors (GSE-3, IE4) on the one hand, and among PHQ-2 and GAD-2 as psychopathology measures on the other hand. The findings further suggest that, within this network of psychopathology on the one hand and resilience measures on the other hand, demoralization seems to be “in between”, as it is neither strongly connected to other psychopathology measures nor to trait resilience measures. Instead, edge weights between demoralization and both depression and anxiety are of similar size as edge weights between demoralization and resilience factors (Fig. 1).

### Unique associations between trait measures and demoralization and depression

Several unique associations between psychopathology and resilience (factors) were observed. The DS-II-Ms showed positive non-zero connections with PHQ-2, GAD-2 and IE4-EC, as well as a negative non-zero connection with IE4-IC. Thus, in this network model, DS-II-Ms was conditionally independent from both GSE-3 and BRS. The edge weight DS-II-Ms – IE4-IC was significantly higher than the edge weights DS-II-Ms – BRS (95% CI 0.05–0.22) and DS-II-Ms – GSE-3 (95% CI 0.02–0.22). In contrast, PHQ-2 was associated with BRS, and to a lower extent, with GSE-3, but conditionally independent from both IE4-IC and IE4-EC . Notably, the edge weight DS-II-Ms – IE4-IC was significantly higher than the edge weight DS-II-Ms – IE4-EC (95% CI 0.14–0.30). The bootstrapped difference tests for all non-zero edge weights and strengths are displayed in Fig. 3.

These results show that among the trait measures (BRS, GSE-3, IE4) only IE4 (more strongly internal than external locus of control) is connected to DS-II-Ms, when controlling for associations with PHQ-2/GAD-2. In contrast, both BRS and GSE-3 (but not IE4) have unique associations with PHQ-2. The findings suggest that demoralization is more strongly linked to generalized perceptions of controllability (IE4) while depression is related to resilience outcome (defined by the perceived ability to recover from stress).

### Network accuracy and stability

**Fig 4:**
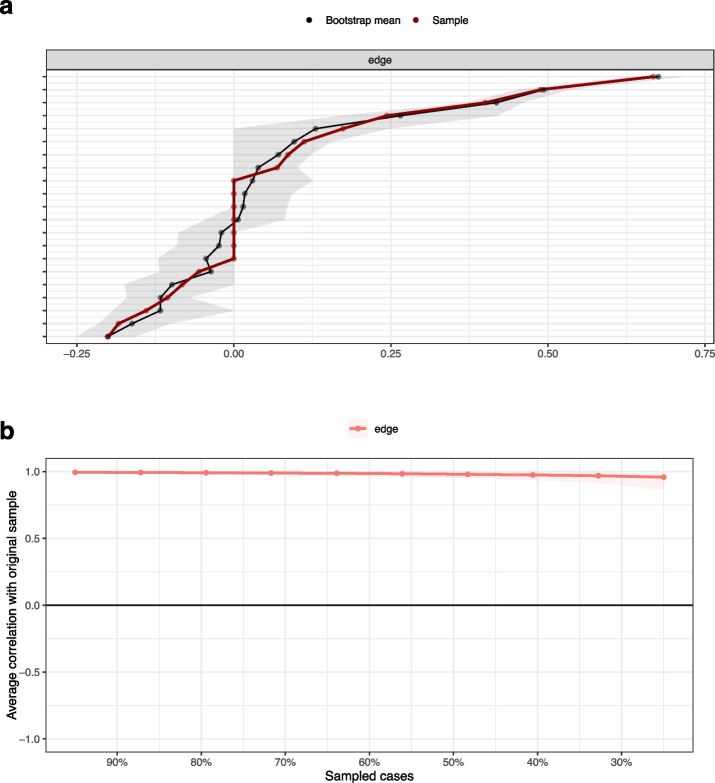
Bootstrapped 95% confidence intervals of the estimated edge-weights, ordered by edge weight (**a**), and average correlations (with 95% CI) between edge weights of networks sampled with persons dropped and edge weights of the original sample (**b**)

Edge weight accuracy (bootstrapped 95% CI) is shown in Fig. 4. A sizeable number of edge weights showed rather narrow bootstrapped confidence intervals, indicating sufficient accuracy.

Tests of stability revealed correlation stability coefficients of 0.75 (for both, edge weights and node strengths), i.e. 75% of the original sample could be dropped, retaining a minimum correlation of *r* =.7 between indices of samples with persons dropped and indices of the original sample, which is well above the recommended cut-off of 0.5 (Fig. 4). Thus, both measures, edge weights and node strengths, represent stable indices within the sample of the German general population.

## Discussion

In this study, we employed a network psychometrics approach to investigate the interrelations between psychopathology measures of demoralization, depression and anxiety, as well as trait measures of resilience, GSE and locus of control, within a representative sample of the general population. The study contributes important evidence on the validity of the DS-II-Ms and the demoralization construct, while providing significant insights into the unique connections between trait resilience measures and psychopathological syndromes.

Regarding the bivariate correlations between demoralization and trait measures, we observed moderate negative correlations with GSE, internal locus of control and resilience, alongside a moderate positive correlation with external locus of control. These results match previous findings, which observed significant relationships between locus of control, GSE and other psychopathological symptom measures including depression, anxiety and stress in general populations [23, 30, 53]. They also add to prior empirical evidence in patients with severe chronic diseases, where moderate negative relationships between demoralization and self-efficacy, resilience and perceived control have been reported [17–20]. The results are consistent with earlier reports on the discriminant validity of depression and anxiety scales [45]. In summary, the correlative results suggest that demoralization as measured by the DS-II-Ms, is distinct from trait measures of resilience, GSE and locus of control, and they further indicate that these trait measures have the expected associations with demoralization.

The observed correlations with depression/anxiety and resilience (factors) are also well in line with theoretical assumptions predicting that repeated negative experiences in dealing with a stressor, a belief that negative events cannot be controlled, and low self-efficacy in coping predispose to developing distress and a state of generalized chronic helplessness, thereby increasing the risk for mental health problems [24, 28, 29, 54].

The network psychometrics approach allowed to further elucidate conditional dependencies between the psychopathological symptoms (depression, anxiety and demoralization) and trait resilience measures. A major finding from this network model was that demoralization showed unique moderate positive associations with both depressive and anxiety symptoms, while, in addition, unique associations between resilience and psychopathology measures occurred. Furthermore, in the network of psychopathology and resilience measures, demoralization was “in between”, being neither strongly connected to depression or anxiety nor to trait resilience measures. First, these findings fit with previous clinical observations showing overlap between demoralization and depression [55, 56]. Second, the result that demoralization has unique associations with resilience factors while only moderately related to other psychopathology measures significantly strengthens the discriminant validity of the demoralization concept. It is in line with previous findings from network studies showing partly distinct symptom clusters of demoralization and depression symptoms in clinical and general populations [12–14]. In previous studies, anhedonia/lack of interest, somatic and cognitive symptoms were most consistently differentiated from demoralization symptoms while in contrast depressive mood (PHQ-2 item) was found to be a key bridge symptom [12, 35, 57]. Moreover, in a previous study, meaningful connections between symptom clusters (“bridges”) were also observed between demoralization and anxiety symptoms [14]. Thus, our conclusion that demoralization reflects a partly distinct phenomenon from related depression and anxiety symptoms is completely in line with recent advances in the field. Furthermore, the result fit to theoretical frameworks, as a sense of helplessness is not only a key symptom of demoralization but also predisposes to depression [28], while both syndromes differ in the way that anhedonia and lack of motivation are absent in demoralized subjects [35].

Another key finding was that we observed unique psychopathology – resilience connections: while depression was associated with resilience and GSE, demoralization was conditionally independent from resilience and GSE but associated with lower internal (and to a lower extent higher external) locus of control. This is an important novel finding, suggesting individuals with higher demoralization rather believe that events are less under their control and are more controlled by powerful others or fate, independently from their perceived self-efficacy and coping abilities. Overall, locus of control as a central correlate of demoralization is in line with the literature, as previous studies have repeatedly shown a crucial role of locus of control in various mental health disorders and mental health in the general population [53, 58, 59]. However, several aspects require consideration. First, while in cancer patients, an external locus of control is related to lower prevalence of depression [60] and less demoralization [61], our network model indicated that, in the general population, an external locus of control is related to adverse mental health outcomes, in particular demoralization. While it might help cancer patients to delegate control to their physicians, overall, an external locus of control seems to be a risk factor, which is in line with prior general population studies [53]. Second, in the present network model, demoralization was more strongly related to internal than to external locus of control, suggesting that the belief that events are under one’s control might be more important to counteract demoralization than a strong belief that events are *not* controlled by powerful others. This finding fits with a previous study in cancer patients, showing internal control was more important than external control in managing perceived threat by the disease [62]. Moreover, the crucial role of an internal control in protecting for demoralization aligns with advances in the neuroscience of the learned helplessness concept, which suggests that it is the presence of control, not the absence of control that is detected by the brain, counteracting a default state of helplessness [63]. Third, there is some prior evidence suggesting that locus of control has no direct impact on mental health outcomes [59]. A cross-sectional study in cancer patients found that locus of control indirectly affects demoralization through coping strategies [61]. In that study, an external locus of control negatively affected demoralization through promoting confrontation with the disease. In contrast, the network model in our study informs that the association between demoralization and locus of control is less dependent from behavior-related experiences in dealing with a stressor (resilience). Indeed, in a previous study, health locus of control was associated with cancer mortality even when adjusting for health-related behavior, suggesting other pathways [64]. In summary, our network model suggests that, in the general population, demoralization could most efficiently be mitigated not by allocating control to powerful others (as in cancer patients) but by strengthening a perception of internal control over events, e.g. through providing adaptive coping strategies, which do not necessarily require the expectation to have the competencies in these strategies nor having successfully implemented these.

Locus of control as well as GSE are conceptually related to the (not clearly defined) construct perceived control. Most frequently, perceived control is defined as negative evaluations of control over negative internal or external stressors. It reflects a well-established transdiagnostic risk factor for mental health issues including depression and anxiety disorders [65, 66]. For instance, perceived control has been observed as a mediator between distress (symptom burden) and depression in somatic diseases [67] and as a predictor of cognitive therapy outcome in anxiety disorders [65]. A recent systematic review investigating cancer patients summarized measures of demoralization, hopelessness and self-efficacy under the concept of perceived control [68]. Our network model generally provides empirical support for this conceptualization, as both demoralization and GSE are uniquely associated with internal locus of control. We thus suggest that the common factor “perceived control” indeed reflects the well-validated concept of locus of control.

### Limitations

Limitations of the study should be recognized. As a cross-sectional study, it does not allow for causal and directional interpretations. Longitudinal studies are required to test whether the observed pathways play a central role for therapeutic applications. Using a network approach, this study illustrated the magnitude of the pathways between psychological variables, which however can only be interpreted within the context of the estimated network model, which includes a selection of variables. The inclusion or exclusion of variables could lead to different results. Nevertheless, the estimated network parameters remained accurate and stable even when dropping a substantial portion of the sample, ensuring that our results are sufficiently reliable for the general population. Although our findings are applicable to the general population, they do not necessarily generalize to specific clinical populations.

### Clinical implications

As described by de Figueiredo, subjects with depression perceive an internal distress, suffer from anhedonia and show a substantial decrease of motivation inhibiting the individual from performing an action, although the direction of this action might be clear. In contrast, the demoralized individual usually has intact consummatory pleasure and sufficient magnitude of motivation, while perceiving uncertainty as to the appropriate direction of action (reflecting the key feature of subjective incompetence [35]). These phenomenological differences are in line with the results from our network model, showing that resilience factors are differentially related to depression symptoms and demoralization. Thus, distinct therapeutic approaches are suggested. Individuals with (specifically) neurovegetative depression need to experience that they have the competencies in performing coping strategies (expectation of self-efficacy) and those strategies were indeed helpful in overcoming a stressor (resilience). Cognitive behavioral interventions including problem-solving techniques support changes on the cognitive as well as the behavioral level and are best suited for treating depression. In contrast, according to our network model, subjects with demoralization would strongly profit from interventions that help to increase the perception of an internal control over distressing external events. This might include both psycho-educative elements informing about adaptive coping strategies in a particular situation and meaning-centered therapies which support better orientation in life.

Furthermore, the network model predicts that, in demoralized individuals, complex cognitive behavioral interventions might be “oversized”, as directly targeting the internal locus of control by offering solutions and new perspectives might be sufficient, not requiring to specifically address the syndrome at the behavioral level. Increasing activity level is instead the key target in depressed individuals, allowing them to increasingly experience themselves to be efficient or successful in overcoming stressors, thereby increasing the magnitude of motivation.

In addition, as our results inform about general population risk factors for demoralization, identifying those at high risk would be the first step in the prevention of a demoralized state. Individuals with adverse childhood experiences, patients with disabling physical diseases and healthy subjects during global crises (e.g. a pandemic) represent vulnerable groups. Thus, early detection of demoralization in high-risk individuals is recommended, which underscores the need to integrate a demoralization screening into psychopathology screenings performed at public health services (e.g. general hospital settings or general practitioners). Individuals with demoralization symptoms could then benefit from group-specific resilience trainings that aim to promote a perception of internal control.

## Conclusions

The study presents an estimated psychological network structure depicting the pathways between central psychopathological symptoms (depression, anxiety, demoralization) and trait measures related to resilience. The pathways through which trait measures of resilience are related to demoralization, depressive and anxiety symptoms can be distinguished, further strengthening the discriminant validity of the demoralization construct. As they represent partly distinct clinical syndromes, demoralization and depression might benefit from different therapeutical approaches. Future longitudinal studies allow to test directional hypotheses between demoralization and resilience factors.

## Supplementary Information

Supplementary Material 1.

## Data Availability

The datasets used and analyzed during the current study are available from the corresponding author on reasonable request.

## Abbreviations

BRS: Brief Resilience Scale
DSM: Diagnostic and Statistical Manual of Mental Disorders, Fifth Edition
DS-II -Ms: Demoralization Scale-II Münster version
EBIC: Extended Bayesian information criterion
GAD-2: General Anxiety Disorder Questionnaire
GSE-3: General Self-Efficacy Short Scale
IE-4: Internal-External Locus of Control Short Scale
PHQ-2: Patient Health Questionnaire
