# Supplementary material for: How demoralization is related to trait resilience factors: a network analysis in a representative sample of the general population
Source: BMC Psychiatry. 2025 Oct 13;25:975. doi: 10.1186/s12888-025-07487-8 (PMC12516848; doi:10.1186/s12888-025-07487-8)
Supplement: Supplementary file 1 — Supplementary Material 1. [file 12888_2025_7487_MOESM1_ESM.pdf]

SUPPLEMENT

	The following statements may apply to you to a greater or lesser extent. For each statement, please indicate the extent to which it applies to you personally.	Does not apply at all	Applies slightly	Applies somewhat	Applies a lot	Applies completely
01	I'm my own boss.	☐	☐	☐	☐	☐
02	If I work hard, I will succeed.	☐	☐	☐	☐	☐
03	Whether at work or in my private life: What I do is mainly determined by others.	☐	☐	☐	☐	☐
04	Fate often gets in the way of my plans.	☐	☐	☐	☐	☐
05	I can rely on my own abilities in difficult situations	☐	☐	☐	☐	☐
06	I am able to solve most problems on my own	☐	☐	☐	☐	☐
07	I can usually solve even challenging and complex tasks well.	☐	☐	☐	☐	☐
08	I tend to bounce back quickly after hard times.	☐	☐	☐	☐	☐
09	I have a hard time making it through stressful events.	☐	☐	☐	☐	☐
10	It does not take me long to recover from a stressful event.	☐	☐	☐	☐	☐
11	It is hard for me to snap back when something bad happens.	☐	☐	☐	☐	☐
12	I usually come through difficult times with little trouble.	☐	☐	☐	☐	☐
13	I tend to take a long time to get over set-backs in my life.	☐	☐	☐	☐	☐

	Over the last two weeks, how often have you been bothered by the following problems?	Not all all	On several days	On more than half of the days	Nearly every day
01	Little interest or pleasure in doing things	☐	☐	☐	☐
02	Feeling down, depressed or hopeless	☐	☐	☐	☐
03	Feeling nervous, anxious or on the edge	☐	☐	☐	☐
04	Not being able to stop or control worrying.	☐	☐	☐	☐

For each of the 16 statements below, please indicate how much (or how strongly) you have felt this way over the last two weeks.

		Never	Sometimes	Often
01	There is little value in what I can offer others.	☐	☐	☐
02	My life seems to be pointless.	☐	☐	☐
03	My role in life has been lost.	☐	☐	☐
04	I no longer feel emotionally in control.	☐	☐	☐
05	No one can help me.	☐	☐	☐
06	I feel that I cannot help myself.	☐	☐	☐
07	I feel hopeless.	☐	☐	☐
08	I feel irritable.	☐	☐	☐
09	I do not cope well with life.	☐	☐	☐
10	I have a lot of regret about my life.	☐	☐	☐
11	I tend to feel hurt easily.	☐	☐	☐
12	I feel distressed about what is happening to me.	☐	☐	☐
13	I am not a worthwile person.	☐	☐	☐
14	I would rather not be alive.	☐	☐	☐
15	I feel quite isolated or alone.	☐	☐	☐
16	I feel trapped by what is happening to me.	☐	☐	☐
